# Visualization of electrographic flow fields of increasing complexity and detection of simulated sources during spontaneously persistent AF in an animal model

**DOI:** 10.3389/fcvm.2023.1223481

**Published:** 2023-09-01

**Authors:** Melissa H. Kong, Steven Castellano, Peter Ruppersberg, Ken S. Lizama, Boaz Avitall

**Affiliations:** ^1^ Ablacon, Inc., Wheat Ridge, CO, United States; ^2^ Deptartment of Medicine, University of Illinois at Chicago, Chicago, IL, United States

**Keywords:** electrographic flow, 3D, mapping, focal activation, arrythmia, atrial fibrillation

## Abstract

**Background:**

Mapping algorithms have thus far been unable to localize triggers that serve as drivers of AF, but electrographic flow (EGF) mapping provides an innovative method of estimating and visualizing *in vivo*, near real-time cardiac wavefront propagation.

**Materials and Methods:**

One-minute unipolar EGMs were recorded in the right atrium (RA) from a 64-electrode basket catheter to generate EGF maps during atrial rhythms of increasing complexity. They were obtained from 3 normal, animals in sinus rhythm (SR) and from 6 animals in which persistent AF which was induced by rapid atrial pacing. Concurrent EGF maps and high-resolution bipolar EGMs at the location of all EGF-identified sources were acquired. Pacing was subsequently conducted to create focal drivers of AF, and the accuracy of source detection at the pacing site was assessed during subthreshold, threshold and high-output pacing in the ipsilateral or contralateral atria (*n* = 78).

**Results:**

EGF recordings showed strong coherent flow emanating from the sinus node in SR that changed direction during pacing and were blocked by ablation lesions. Additional passive rotational phenomena and lower activity sources were visualized in atrial flutter (AFL) and AF. During the AF recordings, source activity was not found to be correlated to dominant frequency or *f* wave amplitude observed in concurrently recorded EGMs. While pacing in AF, subthreshold pacing did not affect map properties but pacing at or above threshold created active sources that could be accurately localized without any spurious detection in 95% of cases of ipsilateral mapping when the basket covered the pacing source.

**Discussion:**

EGF mapping can be used to visualize flow patterns and accurately identify sources of AF in an animal model. Source activity was not correlated to spectral properties of f-waves in concurrently obtained EGMs. The locations of sources could be pinpointed with high precision, suggesting that they may serve as prime targets for focal ablations.

## Introduction

The underlying pathophysiology of atrial fibrillation remains incompletely understood. Efforts to hone in on atrial fibrillation (AF) mechanisms, particularly those contributing to the maintenance and persistence of AF have multiplied and several different methods for identifying these triggers and/or drivers of AF have been put forth using global or panoramic mapping techniques (1–4).

Though reentry in AF was long considered to be the most widely accepted mechanisms of AF and was backed by studies showing spatiotemporal periodicity (5, 6), more recent findings have demonstrated that sustained AF may instead be driven by local foci of variable cycle lengths rather than reentrant circuits (7, 8). Several mapping systems have been developed to identify these areas of repetitive, focal activations during AF, which may also represent targets for ablation to eliminate AF. However, due to the anisotropy of fibrillatory tissue and the chaotic nature of the intracardiac electrograms (EGMs) characteristics of AF, identifying such active focal sources relevant to the initiation and maintenance of AF has remained challenging. A previous study attempting to validate the ability of such mapping systems including Topera (Abbott, St. Paul, MN and Cartofinder (Biosense Webster, Irvine CA) to detect these focal sources found that neither algorithm was able to detect pacing sites as simulated focal drivers of fibrillatory conduction and in fact identified spurious sites as focal and/or rotational activations though they were unrelated to AF induction or maintenance (9).

Electrographic flow (EGF) mapping is an innovative mapping algorithm that enables the full spatiotemporal reconstruction of organized wavefront propagation within the otherwise chaotic electrical conduction of AF and can detect active sources of AF as well as display local passive flow phenomena (10). The theoretical basis and mathematical principles underlying the EGF algorithm have been previously described in detail (11). Using unipolar EGMs recorded from a 64-pole basket mapping catheter, voltage values are transformed into optical intensities with Green’s interpolation and then vector flow fields are estimated based on a modified Horn-Schunck algorithm (11). Accordingly, EGF mapping can create a visual representations of wavefront propagation, measure the level of coherence of these wavefronts, and quantify the activity of sources that serve as drivers of AF. It remains to be determined if calculated source activity (SAC) from these algorithms correlates with more localized bipolar EGMs or if they can accurately identify pacing sites that simulate repetitive focal activations driving AF without displaying epiphenomena.

In this study, we validated the ability of EGF mapping to detect sources of AF. We first demonstrate the use of EGF mapping to visualize a variety of atrial rhythms of increasing complexity in an animal model. Then we compared the unipolar EGMs recorded from a low-density multi-electrode basket catheter with local bipolar EGMs recorded from a high-resolution single point ablation catheter at the location of EGF-identified sources. Finally, we assess of EGF mapping to detect simulated, pacing-induced focal drivers during spontaneously persistent AF in the animal model.

## Materials and methods

### Animal protocol

All procedures were performed in compliance with the Institutional Animal Care and Use Committee (IACUC) guidelines for animal research protocol and were approved by the Animal Care Committee of the University of Illinois at Chicago. Animals (*n* = 9) underwent general anesthesia induced with propofol (7 cc/kg) and maintained with halothane (1%–2%). A duodecapolar catheter (Blazer®DX-20, Boston Scientific, Natick, MA) was advanced from a sheath inserted into the internal jugular into the coronary sinus (CS). In the bilateral femoral veins, 12-Fr short sheaths were introduced for access. Through the 12-Fr sheaths, long steerable sheaths (Agilis™, Abbott, Abbott Park, IL) were inserted to facilitate the advancement of a 50 mm basket mapping catheter with 64 electrodes (AblaCath™; Ablacon, Wheat Ridge, CO and/or FIRMap™; Abbott, Abbott Park, IL) and an ablation catheter into the right (RA) and subsequently left atrium (LA).

### EGF mapping

**Figure 1:**
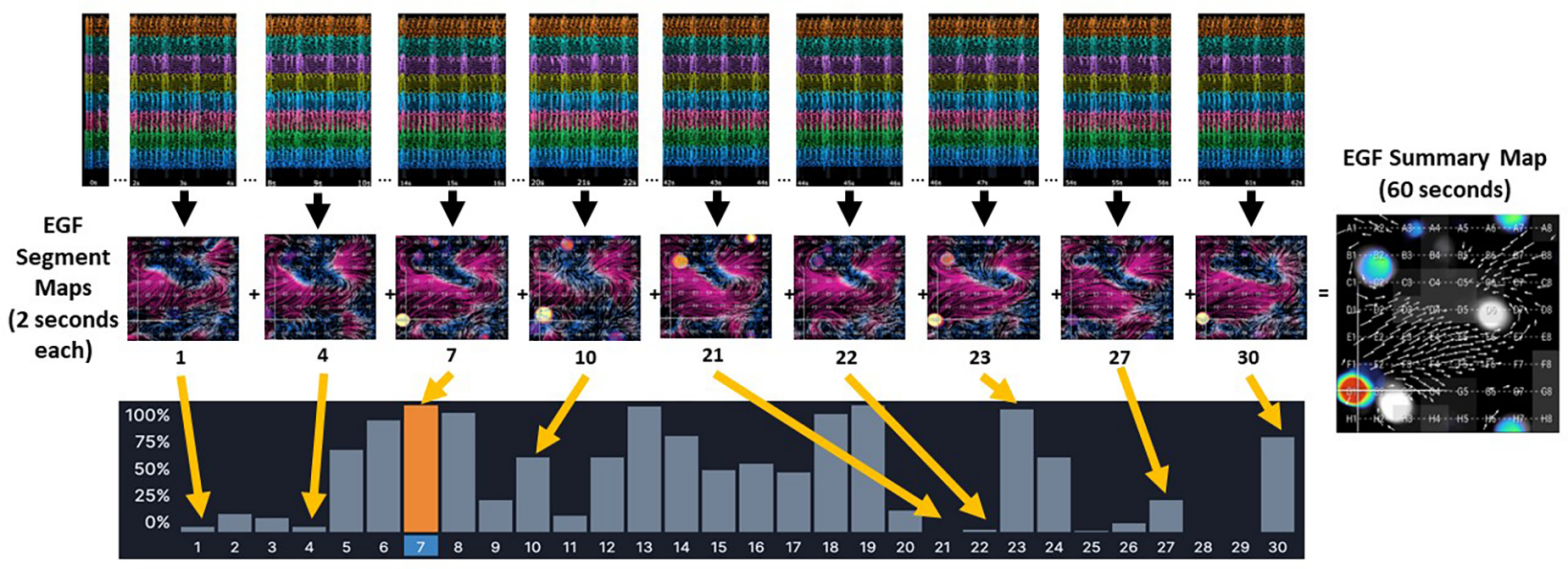
The EGF Summary Map represents a visual aggregation of thirty EGF segment maps, each of which dynamically show flow patterns over a 2 s interval. In the example from a 1 min recording made in an AF animal, there is an active source at G1 (red dot) on EGF Summary Map. However, the time-dependent behavior of this source at G1 can be seen in each individual 2 s EGF Segment Map. In Segment 21 the source at G1 is not active at all (no yellow spot at G1). In Segments 1, 4, 22, and 27, the source at G1 shows low source activity, which is also indicated by the histogram of source activity corresponding to each 2 s segment. In Segment 7, highlighted in orange, the source at G1 is at its most active during any of the 2 s segments recorded.

One-minute recordings were made from standardized positions within the RA, superior vena cava (SVC), SVC/RA, inferior vena cava(IVC)/RA, and RA atrioventricular junction using a 64-electrode basket catheter and the EP Map recording amplifier (EPMap System, Herdecke, Germany). From these 1 min recordings of unipolar EGMs EGF maps were generated. EGF Summary Maps represent a visual aggregation over 1 min of multiple 2 s EGF Segment Maps, which show the time-dependent behaviors of AF sources (Figure 1). Each 2 s EGF Segment Map is calculated by analyzing 105 consecutive 19 msec frames of data. Calculated flow vector fields are analyzed to detect areas in a 3 × 3 pixel window where flow vector angles around a point cover 360° to identify active sources as well as display passive flow phenomenon.

A near field score was also calculated for each electrode by analyzing the ratio of the amplitude of the near field atrial component of the electrograms to the amplitude of the far field ventricular QRST complex. Electrodes were considered “low contact” and the corresponding regions of the EGF maps were flagged whenever this ratio was less than 0.7. The basket was repositioned to achieve greater contact whenever possible.

### EGF mapping and pacing during sinus rhythm

We performed EGF mapping in 3 acute sinus rhythm (SR) animals. For all animals, a 50 mm basket catheter size was selected. Pacing from consecutive electrode pairs along the duodecapolar CS catheter as well as from the ablation catheter was subsequently performed. Pacing was performed in the central RA, RA appendage, SVC sleeve, posterior and lateral RA, near the sinoatrial node, low posterior and anterior RA and in the septum. In addition, to varying the pacing electrodes along the CS and the pacing location within the atria, we systematically varied the pacing output parameters and performed EGF mapping under the following conditions at each location: (1) high output pacing with evidence of local capture; (2) pacing just above capture threshold, again with evidence of local capture; and (3) subthreshold pacing with no evidence of local myocardial capture. In SR, to determine capture threshold, we performed fixed cycle length pacing at a rate that was 10–50 msec faster than the intrinsic sinus rate starting at a pacing output of 20 mA with 2 ms pulse width. In AF, to determine capture threshold, we performed fixed cycle length pacing typically at a cycle length of 50–100 msec. In the AF animals, this rate was required to capture the local myocardium near the electrodes being paced. Pacing output was decremented by 1 mA until loss of capture was seen. Evidence of local tissue capture included change in the activation pattern on the CS catheter, EGM morphology of paced beats v. non-capture beats, and accelerating the cycle length of the local EGMs. The lowest pacing output exhibiting evidence of tissue capture was documented as the capture threshold. High output pacing was defined as pacing at 20 mA with 2 ms pulse width and pacing above capture threshold was defined as pacing at twice the capture threshold output. Subthreshold pacing was typically performed at 1–2 mA with pulse width 2 ms.

In the SR animals, we also performed rapid atrial burst pacing to induce atrial tachycardias, atrial flutters, and in some instances, atrial fibrillation. EGF maps were recorded during any episodes of atrial arrhythmia where at least 1 min could be recorded using the basket mapping catheter.

**Figure 2:**
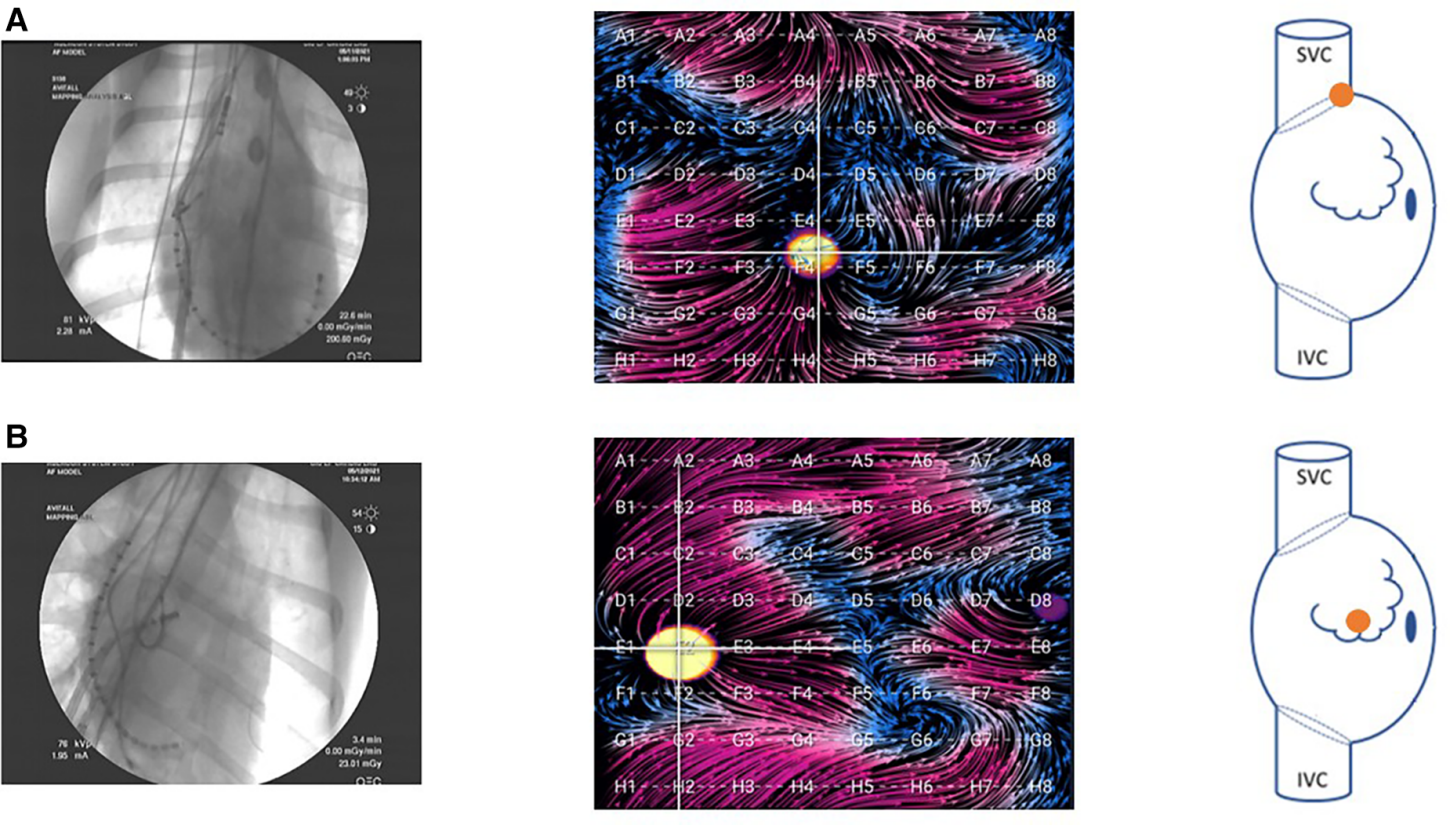
Fluoroscopy images of the basket catheter in (**A**) the SVC-RA junction and (**B**) the central RA. Corresponding EGF maps in these positions are shown during pacing at the location indicated in the accompanying right atrial schematic. In the animal model, these were the primary two positions attained. Due to the small size of the LA in the animal model, there was only one position in this atrium, and the basket could not be fully expanded.

Using an ablation catheter (DiamondTemp™; Medtronic; Minneapolis, MN or Intellatip MIFI™, Boston Scientific, Natick, MA), we performed linear radiofrequency (RF) ablations of the cavotricuspid isthmus (CTI) until a change in atrial activation was seen on the CS catheter and also from the SVC down to the IVC. During each 60 s RF application, EGF maps were recorded. Post-ablation, differential pacing maneuvers were performed. At times aggressive atrial burst pacing was performed after linear ablation to induce atrial arrhythmias. After completing the RA recordings, transseptal catheterization was performed in standard fashion under transesophageal echocardiographic and fluoroscopic guidance with advancement of the ablation catheter and/or 64-pole basket mapping catheter into the LA for pacing and recording within the LA. In the atria, the basket was positioned to optimize deployment/expansion of the splines—this was more easily done in the RA compared with the LA due to the small LA size in the animal model. Example RA positioning is shown in Figure 2.

### EGF mapping and pacing during spontaneously persistent AF

To evaluate EGF mapping during spontaneously persistent AF, an animal model of persistent AF was created using rapid atrial pacing (RAP). Six animals underwent insertion of both an atrial and ventricular pacing wire followed by atrioventricular nodal ablation while under general anesthesia. The ventricular pacemakers were programmed VVI at 90 ppm and the atrial pacemakers were programmed AAI at 650 ppm. Each day, the atrial pacemaker would be inhibited to check the underlying atrial rhythm and once the underlying rhythm was noted to be AF during pacing inhibition, the atrial pacing was discontinued. Rapid atrial pacing was performed until AF was induced and spontaneously persistent in the absence of pacing. Animals were studied after having been in spontaneously persistent AF for greater than 60 days. Similar to the acute SR animals, the 64-electrode basket catheter was inserted for EGF mapping and an ablation catheter and duodecapolar CS catheter were used for ablation and pacing.

### Comparison of unipolar basket EGMs and high-density bipolar EGMs at EGF-identified source locations

In animals with RAP-induced spontaneously persistent AF, spontaneously active sources detected by EGF mapping during AF were localized anatomically using basket coordinates and then interrogated with a high-resolution ablation catheter with 3 tip mini-electrodes (Intellatip MIFI™; Boston Scientific; Natick, MA). The high-resolution ablation catheter was navigated to the endocardial surface nearest the basket electrode closest to the mapped active source. EGF-measured SAC from each 2 s segment of the recording was compared with dominant frequency and f-wave amplitude from the bipolar EGM recordings from the same time segments. Overlapping 4s intervals were employed to robustly sample the recording while minimizing the potential influence of noise or alignment errors and as such consecutive segments had a 2 s overlap with prior segments such that each 60 s recording had 29–30 segments.

### Source simulation and detection during spontaneously persistent AF

In the RAP-induced AF animals, during spontaneously persistent AF, we performed baseline EGF maps and identified any spontaneously occurring active sources of AF. With the 64-pole basket mapping catheter positioned in the RA and LA, pacing was then performed both from the tip of the ablation catheter at a wide variety of ipsilateral and contralateral atrial locations and from various consecutive pairs of electrodes along the duodecapolar catheter positioned within the CS. During AF, pacing output parameters were varied to include high-output pacing; pacing at local capture threshold; and subthreshold pacing to assess the accurate detection of simulated sources without the creation of spurious artifact or false sources. Active sources that were identified during pacing, if present, were localized by the EGF algorithm in each map. A location match was considered to exist when a source was present and either localized to within one electrode distance of the pacing site when pacing was performed with the ablation catheter or to best possible alignment by fluoroscopy when pacing was performed with the duodecapolar catheter.

### Statistical analysis

Summary data are expressed as mean value ± one standard deviation. Two-tailed, two-sample *t*-tests for continuous variables were used to assess differences in dominant frequency, *f* wave amplitude and SAC between two animals. Linear regression was calculated using a line of best fit. The coefficient of determination (*r*^2^) and the *p*-value for the *F*-test of significance of the slope were reported. For all statistical tests, the null hypothesis was rejected at the level of *P* < 0.05.

## Results

### Visualization of electrographic flow fields of increasing complexity

**Figure 3:**
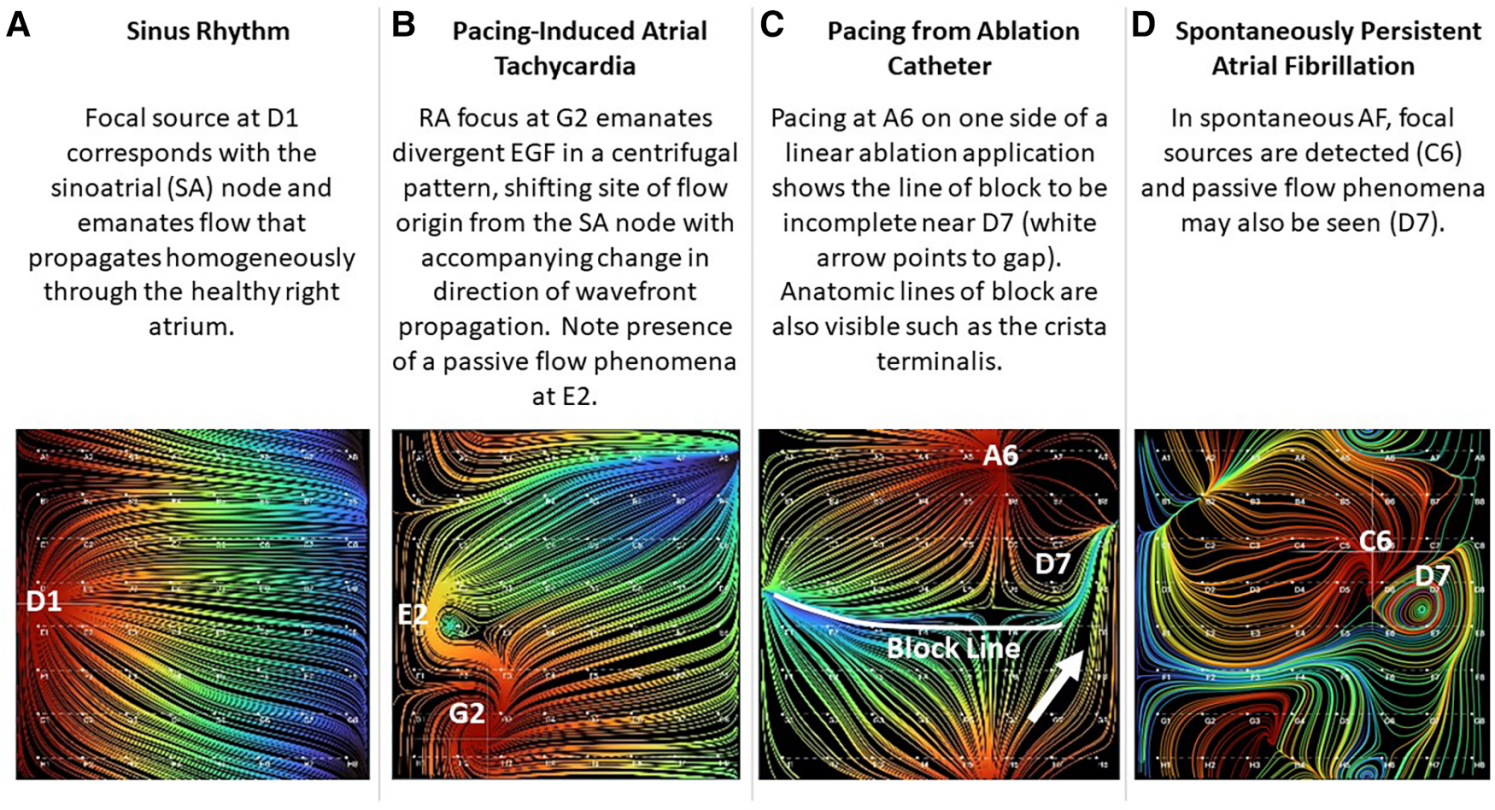
Visualization of electrographic flow fields of increasing complexity.

Three hundred and five sets of EGF Summary Maps and Flow Origin Maps were recorded from SR and RAP-induced AF animals (*n* = 9) during a variety of rhythms. Each rhythm had a characteristic appearance, as shown in the representative EGF maps in Figure 3. In 3 healthy SR animals with mean weight 34.4 ± 2.5 kg, EGF maps recorded with the basket catheter positioned in the central RA during SR revealed focal automaticity from the region of the SA node with homogenous wavefront propagation throughout the healthy RA myocardium (Figure 3A). The origin of flow was at D1, which corresponded to the estimated visual location of the SA node on the electroanatomic map and fluoroscopy while also matching with the site of earliest activation on recording electrodes 19–20 of the duodeca catheter positioned in the SVC/RA junction corresponding to the position of the SA node. As the basket was repositioned, EGF recordings in each location again correctly localized the SA node, the location of which was confirmed by the electroanatomic map, fluoroscopy, and earliest activation on the SVC-RA-CS catheter. EGF mapping of SR was always consistent and reproducible demonstrating origin of flow from the SA node. Additionally, as the 50 mm basket catheter generally filled the RA cavity, addition in SR animals, the crista terminalis was clearly delineated with flow on each side, but not crossing over this anatomic boundary.

**Figure 4:**
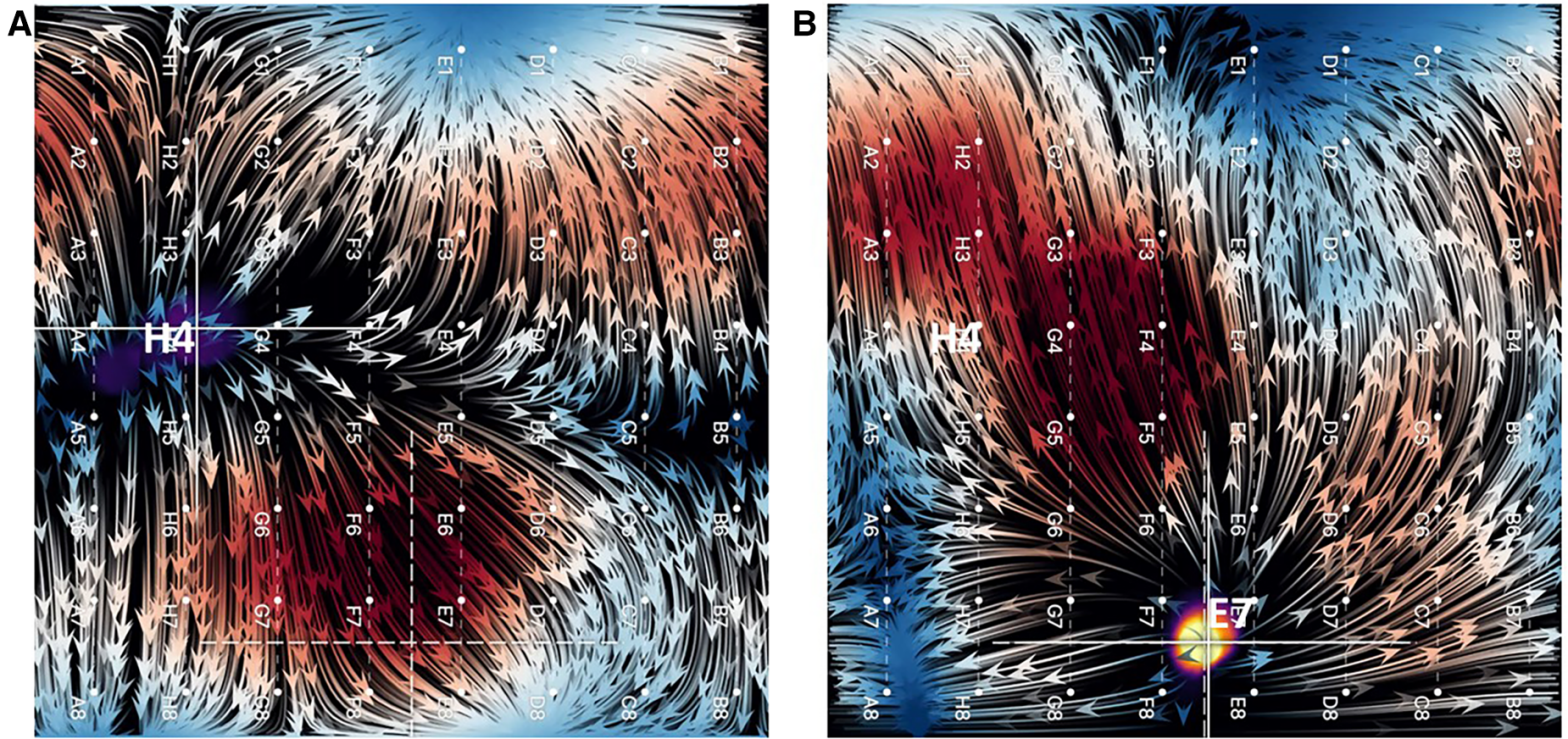
(**A**) EGF map in sinus rhythm with SA node displayed as a focal origin of flow originating from near H4. (**B**) Overdrive pacing of sinus rhythm from tip of ablation catheter positioned near E7. Not only does the focal origin of flow change, but the flow directionality reverses (flow vector arrows).

Similarly, when overdrive pacing of the SA node was performed during sinus rhythm from the tip of the ablation catheter positioned elsewhere in the RA or from electrode pairs on the CS catheter, EGF maps demonstrated an appropriate change in activation pattern, directionality of electrographic flow patterns, and origin of flow from the pacing location whenever pacing was above threshold (Figure 4). This impulse is generally localized to basket coordinates nearest to the pacing electrodes.

Rapid atrial burst pacing in SR animals intermittently resulted in AT post-pacing. EGF maps recorded during pacing-induced AT revealed focal origins of flow at the original location of pacing as well as a change in the direction of wavefront propagation compared with the flow directionality in SR (Figure 3B). During pacing-induced AT, passive flow phenomena could also be observed among otherwise homogenous flow vector lines. Both the active focal source and the passive flow phenomena disappeared after spontaneous conversion to SR. In this particular example shown in Figure 3B, the animal was easily and repeatedly inducible into sustained pacing-induced AT. During recording of AT, the animal degenerated into sustained AF. EGF mapping was repeated during AF and again identified a single active focal source at the original location of pacing. Ablation at the original pacing location resulted in termination of AF back to the SR.

In the SR animals, we also performed a variety of linear ablation lesions including CTI lines and SVC-IVC lines. With pacing from the ablation catheter on one side of a linear ablation application, the line of block could be visualized based on continuity of flow. In Figure 3C, a linear ablation was performed from the SVC to the IVC; however, differential pacing reveals that the line has a gap near D7.

**Figure 5:**
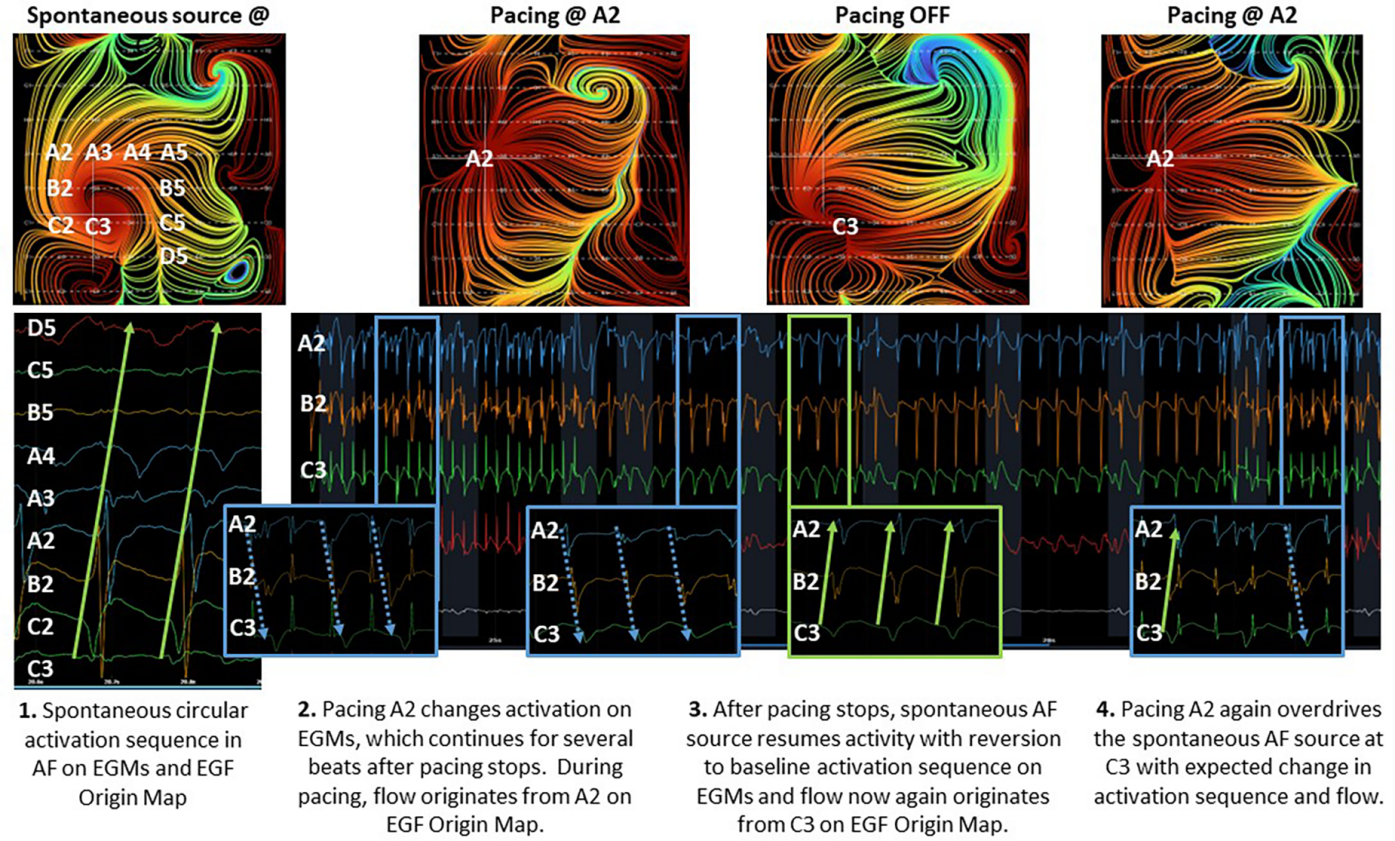
Effect of overdrive pacing on spontaneous AF source.

In 6 RAP-induced AF animals with mean weight 31.4 ± 2.5 kg, rapid atrial pacing was performed for a mean of 18.0 ± 14.9 days until AF was induced. After induction of AF, self-sustained persistent AF continued for a mean of 68.3 ± 15.8 days (Table 1). EGF mapping of spontaneously persistent AF in the RAP-induced AF animals revealed active sources as well as passive flow phenomena that have previously been documented in human AF, such as in the source shown at C6 (Figure 3D). When spontaneous AF sources were detected in the animal model, they could be overdriven with pacing resulting in the expected change in EGF pattern and corresponding change in unipolar EGM activation. Upon cessation of pacing, these EGF patterns reverted back to the pre-pacing activation sequence driven by the spontaneous AF source (Figure 5).

**Table 1:** Baseline characteristics.

Animal (*n* = 9)	Weight (kg)	Duration of RAP to induce AF (days)	Duration of non-induced AF (days)	Total AF duration (days)
SR	37	N/A	N/A	N/A
SR	32	N/A	N/A	N/A
SR	34.1	N/A	N/A	N/A
AF	32.5	14	76	90
AF	35	2	88	90
AF	27.5	8	82	90
AF	30	21	49	70
AF	31.9	45	57	102
AF	31.4	18	58	76
Mean ± SD	32.4 ± 2.78	18.0 ± 14.9	68.3 ± 15.8	86.3 ± 11.5

SD, standard deviation; kg, kilograms; RAP, rapid atrial pacing; AF, atrial fibrillation.

### EGF-identified AF source activity does not correlate with bipolar fractionation

**Figure 6:**
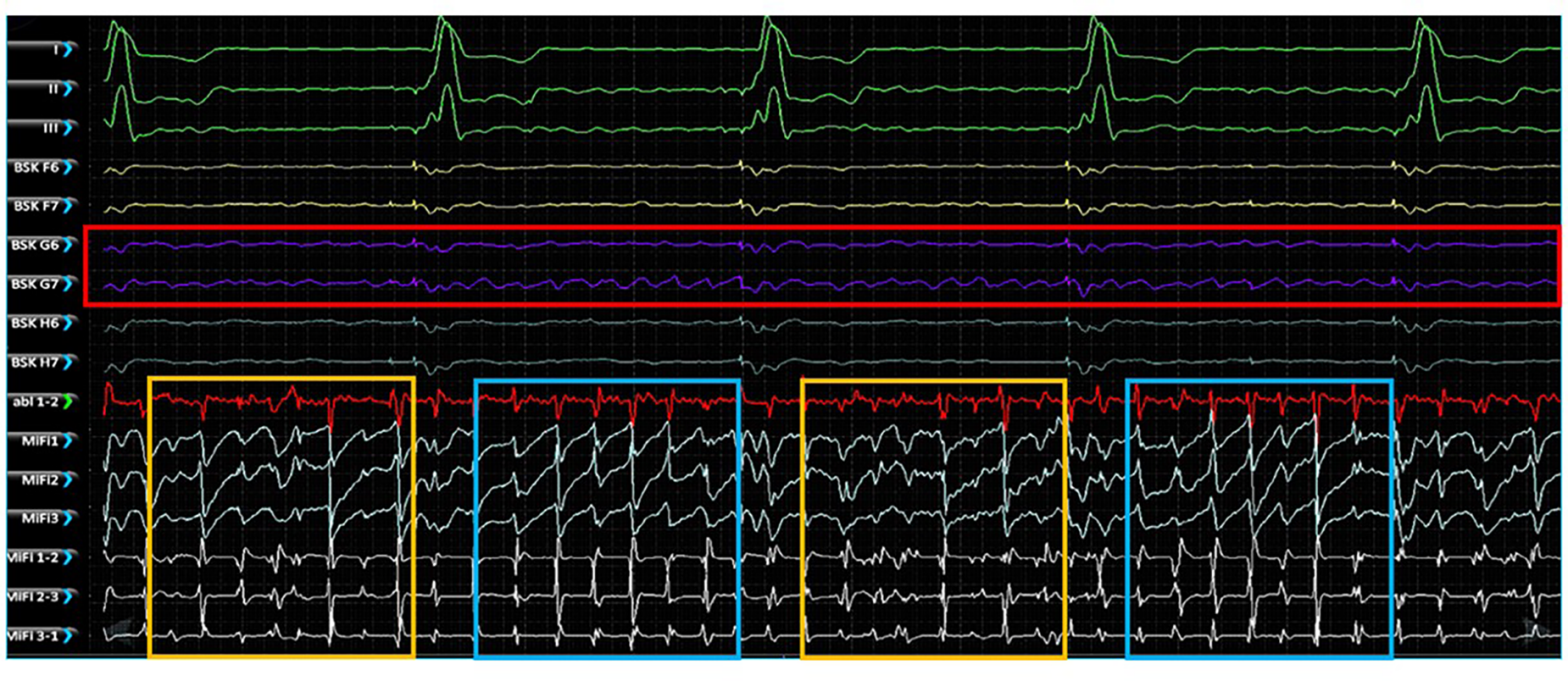
Simultaneous unipolar EGMs recorded from the low-density, lower-contact basket mapping catheter and bipolar EGMs recorded from the high-resolution, contact MiFi ablation catheter positioned at the location of an EGF-identified active source located at G6-7. Basket unipolar EGMs at the G6-7 source location are shown in purple (see red box) with the corresponding unipolar EGMs from each closely spaced ablator tip electrode shown in light blue and bipole pairs shown in white. More organized electrical activity is seen on the high-resolution local ablation catheter electrodes (see blue boxes) interspersed with more chaotic fractionated electrical activity (see yellow boxes). MiFi 1, MiFi 2, MiFi 3 = mini-electrode unipoles; MiFi 1-2, MiFi 2-3, MiFi 3-1—mini-electrode bipoles.

Simultaneous unipolar EGMs from the 64-pole basket catheter and bipolar EGMS from 3 tightly spaced mini-electrodes at the tip of the ablation catheter (MiFi recordings) of 5 EGF-identified active sources were made in 2 animals with RAP-induced spontaneously persistent AF (3 sources in animal A, 2 sources in animal B). At each source, MiFi bipolar recordings demonstrated intermittent salvos of high-frequency, low-amplitude fractionated signal alternating with more organized atrial activity, as shown in the example MiFi recording in Figure 6. However, the unipolar EGMs did not appear to vary in amplitude or frequency in tandem with these alternating patterns of activity.

The MiFi bipolar EGMs were then segmented into overlapping 4 s segments, generating 29–30 such intervals per recording taken at each source. Similar segmentation was performed with the same 4 s intervals of recordings from the 64-unipolar basket EGMs and resulting EGF maps. Dominant frequency and f-wave amplitude were significantly different between the two animals. Dominant frequency was 3.92 ± 0.21 Hz in animal A v. 8.4 ± 2.1 Hz in animal B (*p* < 0.0001), and f-wave amplitude was 0.47 ± 0.04 mV in animal A v. 0.27 ± 0.03 mV in animal B (*p* < 0.0001). SAC was also significantly different between the two animals, but the difference was less pronounced: 15.6% ± 18.2% in animal A v. 26.1% ± 26.8% in animal B (*p* = 0.005). Significant differences in dominant frequency, f-wave amplitude, and SAC among the different sources within each animal were also occasionally present but less distinct.

**Figure 7:**
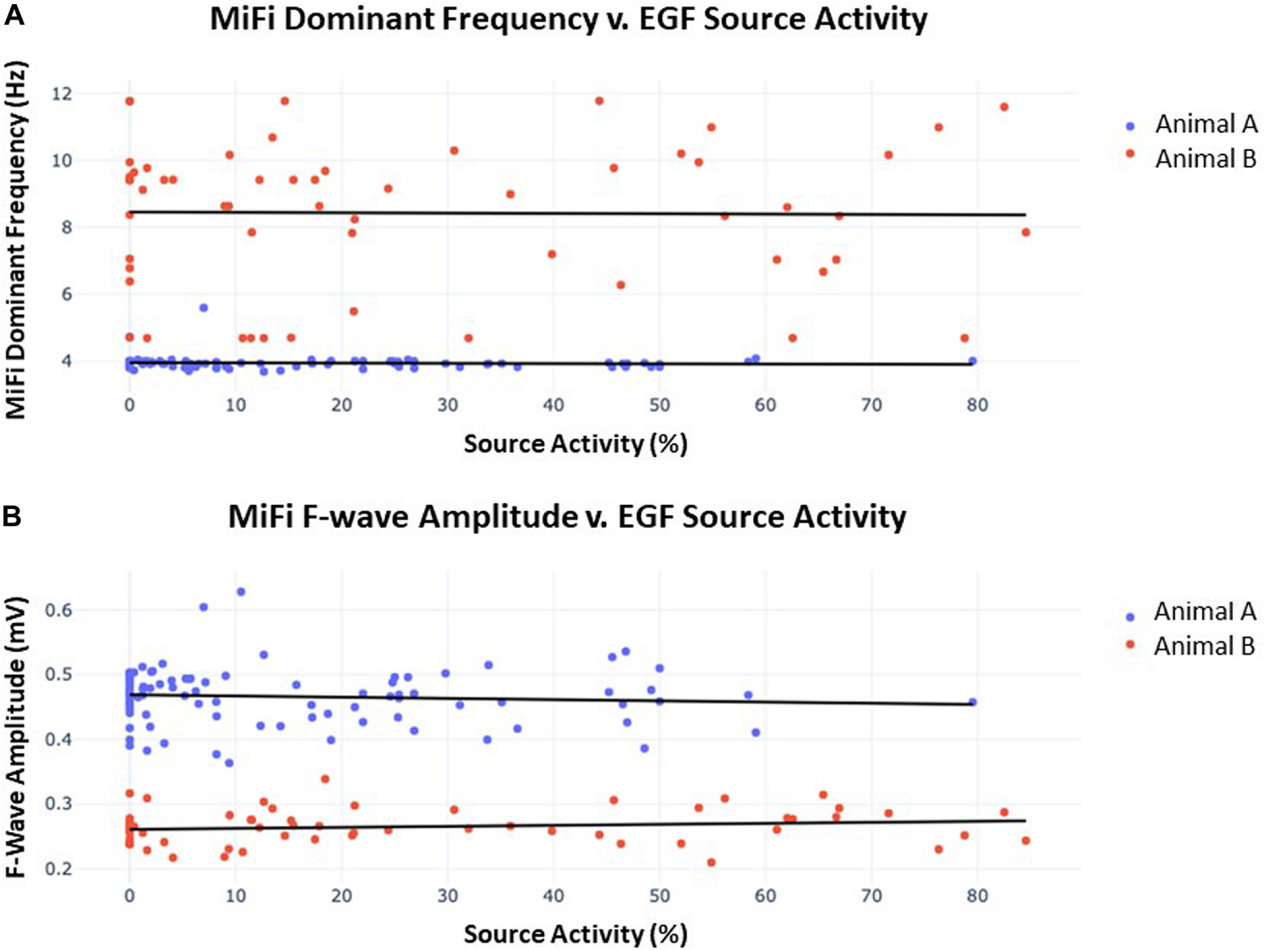
(**A**) Dominant frequency as measured by MiFi catheter v. concurrently-obtained EGF-quantified source activity. 60 s recordings were obtained from each of 3 sources in animal A and 2 sources in animal B. Each datum represents a 4 s interval with 2 s overlap to the proceeding segment such that 29–30 intervals were generated per recording. A line of best-fit was then plotted for each animal. In animal A, *r*^2 ^= 0.003 and *p* = 0.59, similar to *r*^2 ^= 0.001 and *p* = 0.92 in animal B. There were no significant differences in dominant frequency across individual sources within each animal. (**B**) F-wave amplitude measured by MiFi catheter v. concurrently-obtained EGF-quantified source activity. Data was obtained as described in (**A**) from the same 5 sources. In animal A, *r*^2 ^= 0.006 and *p* = 0.46, similar to *r*^2 ^= 0.022 and *p* = 0.26 in animal B. There were no significant differences in *f*-wave amplitude across individual sources within each animal.

Despite these trends, there was ultimately no correlation between the time-synchronized SAC and local fractionation as determined by the dominant frequency or f-wave amplitude. Figure 7 shows the scatter plot of SAC v. dominant frequency and SAC v. f-wave amplitude of the local bipolar EGMs across all 4 s intervals of recording time in both animals (*n* = 89 and *n* = 60, respectively). A line of best-fit for SAC vs. dominant frequency resulted in *r*^2 ^= 0.003, *p* = 0.59 for animal A and *r*^2 ^= 0.001, *p* = 0.92 for animal B, while a line of best-fit for SAC vs. f-wave amplitude resulted in *r*^2 ^= 0.006, *p* = 0.46 for animal A and *r*^2 ^= 0.022, *p* = 0.26 for animal B. There were similarly no significant correlations across the EGF and bipolar EGM metrics in any of the 5 sources when data was segregated by source.

### EGF mapping algorithm can accurately detect simulated sources of AF in an animal model

In the 9 animals studied, we performed 78 recordings during pacing: in 41 of these recordings, the basket was recording from the same chamber being paced. In 37 of these recordings, the basket was in the opposite chamber from the site of pacing. Forty-four of the pacing experiments were performed at or above local capture threshold during AF.

**Figure 8:**
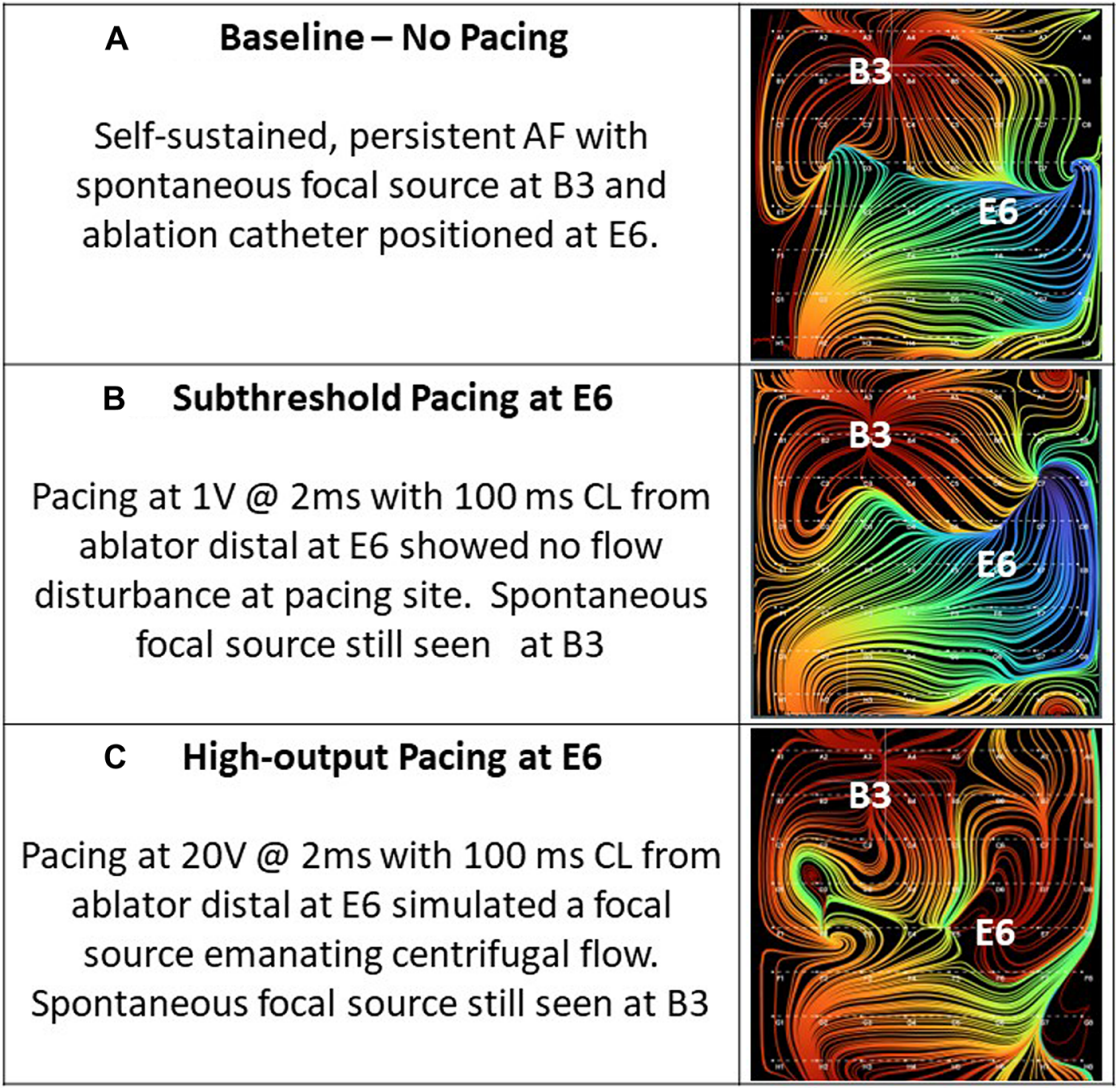
(**A**) Baseline—no pacing. Self-sustained, persistent AF with spontaneous focal source at B3 and ablation catheter positioned at E6. (**B**) Subthreshold pacing at E6. Pacing at 1 mA @ 2 ms with 100 ms cycle length from ablator distal at E6 showed no flow disturbance at pacing site. Spontaneous focal source still seen at B3. (**C**) High-output pacing at E6. Pacing at 20 mA @ 2 ms with 100 ms cycle length from ablator distal at E6 simulated a focal source emanating centrifugal flow. Spontaneous focal source still seen at B3.

Thirty-four of the recordings were taken during subthreshold pacing, which again resulted in 0% detection of a simulated source. Of the remaining recordings taken during pacing at or above capture threshold in the ipsilateral atrium, 21 of 21 (100%) recordings accurately localized the simulated focal source with only 1 of 21 (4.8%) showing artifact. This artifact occurred during high-output CS pacing near the SVC. Figure 8, shows example RA EGF maps without pacing (Figure 8A), unchanged during subthreshold pacing in the RA (Figure 8B), and with a new focal source detected during pacing at or above threshold in the RA (Figure 8C). By contrast, pacing at or above threshold in the opposite atrium from the basket resulted in no maps showing a simulated source and only 1 of 23 (4.3%) recordings showing any shift in flow pattern at all. This shift occurred during high-output (10 V) pacing in the LA at the RSPV and resulted in a shift in the flow pattern near the CS of the RA map.

## Discussion

The primary findings of this study were (1) EGF mapping can panoramically visualize atrial flow fields of increasingly complex electrical phenomena; (2) EGF mapping efficiently identifies sources of electrical activity that drive AF while local fractionation is not useful when it comes to understanding the global atrial electrical activity; and (3) during a wide variety of pacing experiments, simulated focal sources were reliably detected without false positive source detections or epiphenoma. By observing EGF recordings in various rhythms, we demonstrate that EGF provides an intuitive representation of wavefront propagation in near real-time. The parallel arrangement of closely spaced flow lines in SR supports that SR flow is high in magnitude and unidirectional. Furthermore, the ability to accurately track the SA node as the basket was repositioned lends support to the accuracy of the EGF mapping algorithm and flow field visualization despite the use of a low-density basket mapping catheter.

While SR recordings showed high coherence of activity originating from the SA node, more complex rhythms sometimes revealed passive flow phenomena and competing, but lower activity sources that created more chaotic flow patterns. These phenomena were again physiologically significant with focal sources localizing to the pacing site when pacing was performed at or above threshold. In retrospective analysis, EGF-identified active sources appear to have clinical relevance as their presence at the end of an ablation procedure predicts recurrent AF post-ablation (12). The prospective, multi-center, randomized controlled *FLOW-*AF trial (NCT04473963) has also now been completed and enrolled 85 persistent and long-standing persistent AF patients (13). The results of this trial were presented and are pending publication, but showed that EGF-identified, extra-pulmonary vein sources were detected in 60% of a difficult-to-treat redo population of non-paroxysmal AF patients after undergoing successful Pulmonary Vein Isolation (PVI). These sources were successfully eliminated 95% of the time with resultant changes in flow characteristics and the ablation of these sources after PVI resulting in a 51% improvement in AF-free survival on an absolute basis at 1 year post-procedure (14).

Time-dependent dominant frequency and f-wave amplitude during AF can be recorded with MiFi electrodes colocalized to basket locations of EGF-identified sources; however, it does not correlate with the dynamic broader atrial electrical activity identified by EGF mapping as AF source activity. This finding confirms the unlikely contribution of fractionated activity alone as a driver of AF or as an ablation target. Although dominant frequency, *f* wave amplitude and SAC were all significantly different between the two animals (*p* < 0.01), dominant frequency and f-wave amplitude ultimately did not correlate with time-matched SAC (*p* > 0.1). Metrics extractable from local bipolar EGMs therefore did not seem to relate to the broader dynamic electrical properties obtained from EGF mapping, suggesting that an understanding of the latter is needed to fully comprehend flow properties.

Since dominant frequency and f-wave amplitude instead varied most from animal-to-animal rather than from segment-to-segment, these metrics might in fact relate more to intrinsic properties of an animal's cardiac substrate than to temporal changes in rotor properties. After all, animal A had sources located more laterally in the RA as well as a lower dominant frequency when compared to animal B, so this interpretation also concurs with past studies showing that the spectral properties of fibrillatory electrograms varied most from moving laterally across the atria. Such a relationship is believed to be a consequence of the left-to-right gradient in the inward rectifier potassium current (15, 16) and has even been shown to result in *f* wave properties being similar at a given location regardless of if fibrillatory conduction led to AF induction or not (9).

Lastly, we demonstrated that EGF mapping can accurately detect pacing sites simulating focal drivers of AF 100% of the time with only 5% additional spurious activity observed. Such results improve greatly on those of other mapping systems, which failed to localize fibrillatory conduction to the pacing sites and instead localized to spurious sites that actually could not have been sources of activation in the presence of pacing without AF induction (9). By contrast, EGF only rarely identified potentially erroneous source locations not aligned with the pacing site. Only 4% of maps obtained during pacing-induced AF at or above threshold in the contralateral atrium had any changes in their EGF patterns after pacing. Moreover, no maps revealed any new sources or changes in flow patterns resulting from subthreshold or contralateral pacing, indicating that non-propagating electrical activity does not mimic a source or otherwise interfere with flow lines. Similarly, source detection did not continue once pacing was terminated, suggesting a temporal responsiveness to source presence.

Ultimately, the observations that EGF mapping localized sources accurately but only when the basket electrodes adequately covered the endocardium where the pacing was located indicate that EGF mapping is a clinically valuable tool for detecting true drivers of AF propagation without identifying irrelevant flow phenomena that do not contribute to the initiation and/or maintenance of AF. Its accuracy in detection of simulated focal sources suggests that the EGF algorithm can correctly localize extra-PV sources as individually tailored ablation targets.

## Limitations

The primary limitation of this study is the small number of animals studied. While the RAP-induced AF animal model may not be mechanistically the same as clinical AF in humans, source characteristics and behaviors seen in the animal model correspond with source characteristics and behaviors that have been documented in humans. Manipulation of even the smallest, 50 mm basket mapping catheter within the animal atria—particularly in the LA—was more challenging and in many cases the basket was constrained by the chamber size, which may affect spatial interpolation.

## Conclusion

EGF mapping using a panoramic basket catheter allows for the visualization of cardiac flow propagation in an interpretable manner. There was no time-varying correlate of EGF-measured SAC identified from local bipolar EGMs, confirming the need for broader mapping of electrical activity throughout the atrium rather than at source locations alone. EGF maps could also accurately identify pacing sites as drivers of AF without detecting spurious sites, which in turn allows them to define targets for ablation.

## Data Availability

The datasets presented in this article are not readily available because map data is generated using a proprietary algorithm. Requests to access the datasets should be directed to Boaz Avitall; bavitall@uic.edu.
